# Systematic review and meta-analyses of intensity-modulated radiation therapy versus conventional two-dimensional and/or or three-dimensional radiotherapy in curative-intent management of head and neck squamous cell carcinoma

**DOI:** 10.1371/journal.pone.0200137

**Published:** 2018-07-06

**Authors:** Tejpal Gupta, Sadhana Kannan, Sarbani Ghosh-Laskar, Jai Prakash Agarwal

**Affiliations:** 1 Department of Radiation Oncology, ACTREC/TMH, Tata Memorial Centre, Homi Bhabha National Institute (HBNI), Mumbai, India; 2 Clinical Research Secretariat (CRS), ACTREC, Tata Memorial Centre, HBNI, Navi Mumbai, Maharashtra, India; Northwestern University Feinberg School of Medicine, UNITED STATES

## Abstract

**Introduction:**

Technological advancements in treatment planning and delivery have propelled the use of intensity-modulated radiation therapy (IMRT) in head and neck squamous cell carcinoma (HNSCC). This review compares IMRT with conventional two-dimensional (2D) and/or three-dimensional (3D) radiotherapy (RT) in curative-intent management of HNSCC.

**Methods:**

Only randomized controlled trials (RCTs) offering curative-intent RT in patients with non-metastatic HNSCC were included. Outcome data was extracted independently by two reviewers, pooled using the Cochrane methodology, and expressed as risk ratio (RR) or hazard ratio (HR) as appropriate with 95% confidence intervals (CIs). Xerostomia was the primary outcome of interest whereas loco-regional control, overall survival and quality-of-life (QOL) were secondary endpoints.

**Results:**

Seven RCTs involving 1155 patients directly comparing IMRT with 2D/3D-RT in HNSCC were included. The primary objective in five of seven index RCTs was reduction in xerostomia, with only one trial each using loco-regional control and overall survival as primary endpoints for sample size calculation. The use of IMRT was associated with a 36% relative risk reduction in ≥grade 2 acute xerostomia (RR = 0.64, 95%CI = 0.49–0.84; p = 0.001) compared to 2D/3D-RT. More importantly, IMRT significantly reduced the risk of ≥grade 2 late xerostomia (RR = 0.44, 95%CI = 0.34–0.57; p = 0.00001) compared to non-IMRT techniques at all time-points. Within the limitations of inadequate sample size and low statistical power, IMRT also resulted in 24% relative reduction in the risk of loco-regional relapse (HR = 0.76, 0.57–1.01; p = 0.06) and 30% relative reduction in risk of death (HR = 0.70, 95%CI = 0.57–0.88; p = 0.002) compared to 2D/3D-RT. However, this benefit of IMRT for loco-regional control and overall survival was limited to nasopharyngeal cancer patients alone, with no significant difference in efficacy between the two techniques in patients with cancers of the laryngo-pharynx in this analysis, highlighting the inconsistency in results of subgroup analyses stratified by primary site. Inadequate reporting of data precluded statistically pooling of results for QOL outcomes.

**Conclusions:**

There is consistent moderate-quality evidence that IMRT significantly reduces the risk of moderate to severe acute and late xerostomia compared to 2D/3D-RT in curative-intent radiotherapeutic management of HNSCC. However, the quality of evidence regarding the superiority of IMRT over conventional techniques for disease-related endpoints is rather low due to relative lack of power and inconsistency of results precluding robust conclusions.

## Introduction

Radiotherapy (RT) combined with concurrent systemic chemotherapy as appropriate, is the contemporary standard of care in the curative-intent management of head and neck squamous cell carcinoma (HNSCC), both in the definitive, non-surgical as well as post-operative adjuvant setting [1,2]. Traditionally, in the olden days (before the conformal era), head-neck cancers were treated with conventional RT techniques typically comprising of either a set of parallel opposed portals with or without matched low anterior neck field or a wedge pair portal based on two-dimensional (2D) fluoroscopic imaging without major emphasis on shielding normal tissues [2] resulting in considerable acute and late morbidity [3,4]. Common acute toxicity of head-neck irradiation includes mucositis, dermatitis, dysgeusia, dysphagia, and odynophagia resulting in inadequate oral intake and consequent weight loss, which sometimes can lead to interruption and even premature discontinuation of therapy with potential adverse impact on outcomes [2,3]. The most common debilitating late toxicity is radiation-induced xerostomia (dry mouth) caused by salivary gland hypofunction leading to persistent dryness of mouth, oral discomfort, and difficulty in speech and swallowing [4,5]. There is consistent evidence that xerostomia has a negative impact upon health-related quality-of-life (QOL) in long-term survivors of head-neck cancer [5,6]. Over the years, technological advances in treatment planning and delivery based on three-dimensional (3D) computed tomographic (CT) imaging have resulted in progressive conformation [2] of radiation dose to the target tissues while sparing adjacent organs-at-risk (OARs). Intensity-modulated radiation therapy (IMRT) defined as an advanced form of high-precision conformal technique using non-uniform beam intensities determined through computer-based optimization to achieve the desired dose-distribution, has emerged as the most preferred technique [7] and has been readily adopted by the head and neck oncology community worldwide in the curative-intent radiotherapeutic management of HNSCC.

Notwithstanding the cost and complexity, IMRT quickly supplanted older radiation techniques (2D-RT/3D-RT) with its promise to improve the therapeutic index based on dosimetric comparison [8], single-institution prospective studies [9,10] and multi-centric co-operative group trials [11–13]. In the last decade, several randomized controlled trials (RCTs) have directly compared IMRT with either 2D-RT or 3D-RT for various sites in the head and neck. While nearly all trials reported significant reduction in moderate to severe xerostomia with IMRT, impact upon tumor and survival has been inconsistent, possibly due to small sample size and associated low statistical power of individual studies. Statistical pooling of data from individual studies using modern meta-analytic methods is a common tool to circumvent some of these limitations and generate high-quality evidence. An earlier meta-analysis [14] of 5 RCTs [15–19] concluded that the use of IMRT for HNSCC was associated with a significant reduction in grade 2–4 xerostomia without compromising loco-regional control (LRC) or overall survival (OS). However, the authors did not perform any subgroup analyses stratified by either the technique of irradiation (2D-RT and 3D-RT) or the site of primary tumor (nasopharynx and laryngo-pharynx). In addition, their analysis was restricted to the index publications with no attempt to update any trial data. Efficacy outcomes (LRC and OS) were extracted and pooled only from 3 of 5 available studies. Recently, 2 more trials [20,21] randomly assigning patients with laryngo-pharyngeal cancers to either IMRT or 3D-RT have been reported with extractable data for relevant endpoints prompting the conduct of the present updated systematic review and meta-analyses. The primary aim of this systematic review and meta-analysis was to compare IMRT with conventional RT (2D/3D-RT) in curative-intent radiotherapeutic management of HNSCC using xerostomia, LRC, OS, and QOL as outcome measures.

## Methods

This systematic review was carried out in accordance with the Cochrane handbook [22] for systematic reviews of interventions. Quality of evidence was appraised and graded using the Grades of Recommendation, Assessment, Development, and Evaluation (GRADE) system [23] and reported using the Preferred Reporting of Systematic Reviews and Meta-Analyses (PRISMA) guidelines [24].

### Literature search strategy

Eligible studies directly comparing IMRT with either 2D-RT or 3D-RT in the radiotherapeutic management of HNSCC were identified through a systematic search of the medical literature using a validated search strategy. An electronic search of Medline via PubMed was conducted from January 1995 onwards till May 2017 with no language or publication status restrictions. The Cochrane Central Register of Controlled Trials (CENTRAL) and Database of Abstracts of Reviews of Effectiveness (DARE) were also searched electronically from inception till May 2017. Details of the search strategy are presented separately (S1 Appendix). Electronic search was further supplemented by hand-searching of review articles, cross references and conference proceedings.

### Study selection

For inclusion in the meta-analysis, trials had to be RCTs, include previously untreated patients with non-metastatic HNSCC, offer curative-intent treatment either in the definitive or post-operative adjuvant setting, and not be confounded by additional therapeutic differences between the two groups. Trials using brachytherapy boost in addition to external beam RT were also included, provided the boost was offered in both arms. Trials using chemotherapy (induction, concurrent, or adjuvant) were also considered eligible provided the chemotherapy regimen (drugs, dosage, scheduling) was identical in both arms. For trials with more mature data published or presented at a date later than the index publication, relevant data was also extracted from the update.

### Data extraction

Two reviewers (TG and SK) independently extracted relevant data from individual studies with discrepancy, if any, being resolved by consensus. Xerostomia was the primary outcome of interest while LRC, OS, and QOL were secondary endpoints. Outcome data was extracted and pooled using the Cochrane methodology [22] for meta-analysis using the fixed-effects or random-effects model as appropriate and expressed as risk ratio (RR) or hazard ratio (HR) with respective 95% confidence intervals (CIs). Trials were subgrouped appropriately according to RT technique (2D-RT or 3D-RT) for toxicity outcomes (xerostomia) and site of primary (nasopharynx or laryngo-pharynx) for efficacy outcomes (LRC and OS). In addition, analysis for late xerostomia was done at different time-points (6-month, 1-year, 2-year and 3-year) for better interpretation and informed decision-making. The analysis, interpretation, and reporting of results also included a risk of bias assessment [22] for all included individual studies and grading [23] of the strength of recommendation. All analyses were done using Review Manager (RevMan) version 5.3 and GRADE profiler (GRADEpro) version 3.6.1 (The Nordic Cochrane Centre, Cochrane Collaboration, 2008).

## Results

**Fig 1:**
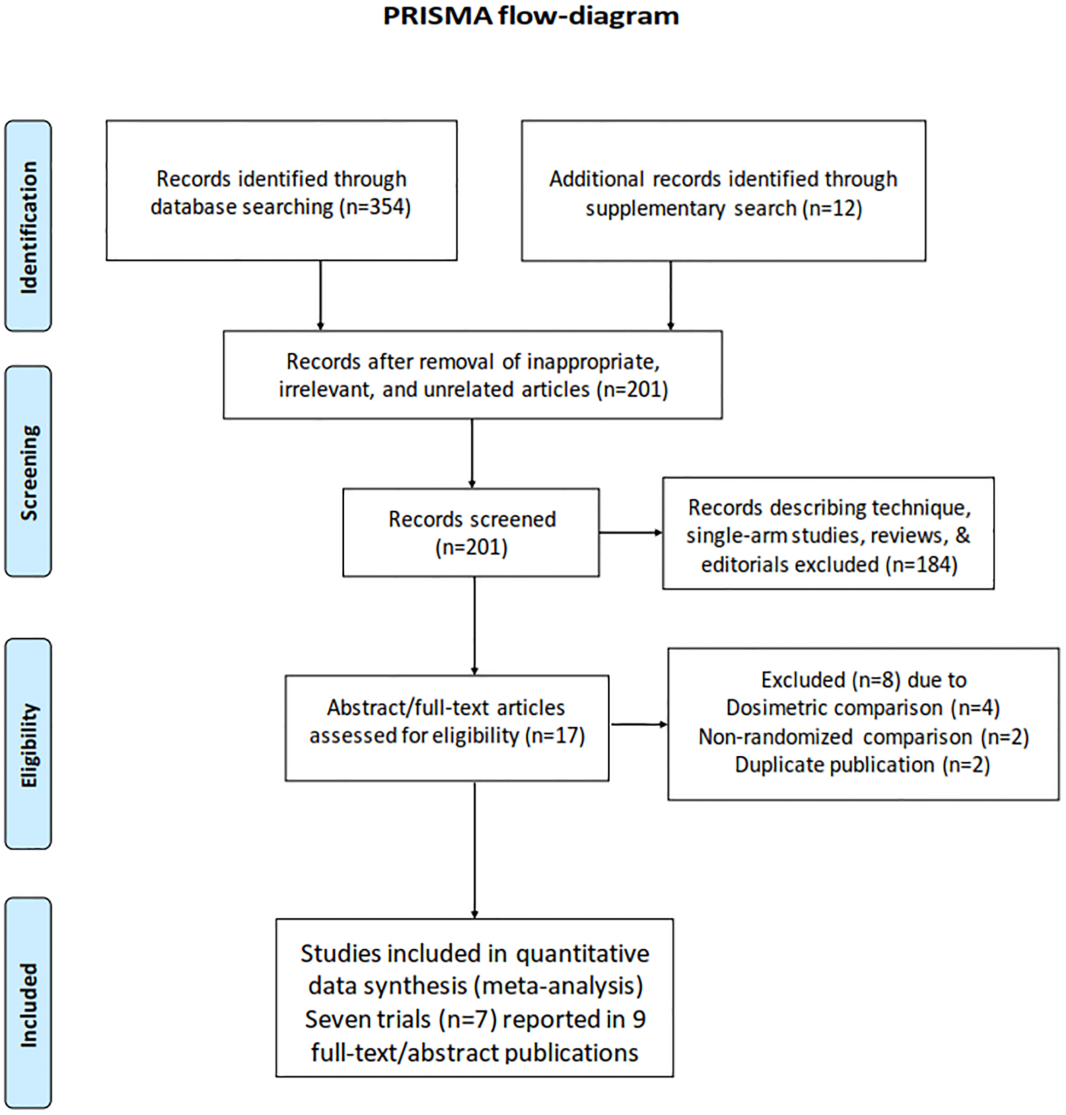
Flow-diagram of study selection and inclusion in the systematic review and meta-analyses as per PRISMA guidelines.

The flow-diagram of study selection and inclusion in the meta-analysis as per the PRISMA guidelines [24] is depicted in Fig 1. Comprehensive and systematic search of the medical literature using the described search strategy identified 366 records that were retrieved for further review. Large number of these records (n = 165) were considered inappropriate, irrelevant, or unrelated leaving 201 abstracts that were screened. Of these, 192 abstracts were excluded (reports describing radiotherapy technique, single-arm studies, review articles, editorials, dosimetric comparisons, non-randomized comparative studies, and duplicate publication) leaving a total of 9 abstracts, wherein full-text articles were retrieved wherever available for consideration for inclusion in the meta-analyses. One study [15] published as full-text earlier was subsequently updated with more mature results for disease-related outcomes through an abstract [25]; appropriate data from both was included in the meta-analyses. One study reported QOL data separately [26] from the results of the index RCT [18], which was also included in this systematic review. For the most recent trial [21], data was extracted from the abstract and conference presentation pending full-text publication. Finally, 7 prospective RCTs involving a total of 1155 unique patients comparing IMRT versus 2D-RT/3D-RT in curative-intent radiotherapeutic management of HNSCC were included for data extraction and evidence-synthesis in the meta-analysis.

### Description of included studies

Treatment characteristics and clinical outcomes of patients included in the 7 RCTs directly comparing IMRT with 2D-RT/3D-RT are summarized in Tables 1 and 2 respectively. Salivary gland toxicity (xerostomia) was the primary endpoint for 5 of the 7 included RCTs [15–18,20], and secondary endpoint [19,21] in two of them. Only a single study each used LRC [21] and OS [19] as primary endpoints. Assessment of salivary gland toxicity was variable and heterogeneous in terms of scoring criteria and time-points of assessment. One study [15] assessed salivary function by stimulated whole salivary flow-rates and defined severe toxicity as post-RT salivary flow <25% of pre-RT flow [27]. Another study [19] reported radiation toxicity including xerostomia by the Common Toxicity Criteria (CTC) version 3.0 [28]. Five studies [16–18,20,21] reported xerostomia using the Radiation Therapy Oncology Group (RTOG)/European Organization for Research and Treatment of Cancer (EORTC) morbidity criteria [29], while one study [17] primarily used the Late Effects on Normal Tissue/Subjective Objective Management Analytic (LENT SOMA) scale [30,31]in addition to the RTOG criteria. Five of 7 studies were rather small comprising of less than 100 patients (in both arms). Only two studies, one from China that included over 600 patients with nasopharyngeal cancers [19] and the other from France [21] led by Groupe d’Oncologie Radiotherapie Tete Et Cou (GORTEC 2004–01) with a planned accrual of over 300 patients with oro-hypopharyngeal cancers were adequately powered for disease-related outcomes. Unfortunately, GORTEC 2004–01 study [21] had to be terminated prematurely (after randomizing 188 patients) due to slow accrual and adoption of IMRT as standard treatment in France. Three studies [15,16,19] were restricted to patients with nasopharyngeal cancer while the remaining 4 studies [17,18,20,21] included patients with non-nasopharyngeal sites such as oropharynx, hypopharynx, larynx, and oral cavity. Four studies used 2D-RT as the control arm [15–17,19], while 3D-RT was the control arm in 3 studies [18,20,21]. QOL was assessed by the EORTC general Quality-of-Life Questionnaire (QLQ-C30) and specific Head Neck (HN35) module in 4 studies [15,17,18,20] and a customized xerostomia questionnaire (XQ) in one study [16]. One study each used Medical Outcomes Short Form (SF36) [15] and modified XQ [17] in addition to EORTC QOL questionnaires for additional information on patient-reported outcomes.

**Table 1:** Characteristics of studies included in the systematic review and meta-analyses.

Author (year)	Site(s)	Stage	Randomization	Primary endpoint	Criteria/Methods	QOL assessment(s)
Pow (2006) *Kwong (2008)	Nasopharynx	T2, N0-N1 (stage II)	IMRT: 42	Change in stimulated whole salivary flow-rate at 1-year	Post-RT salivary flow <25% of pre-RT flow	Short Form (SF) 36 and QLQ C30 & HN35
			2D-RT: 40			
Kam (2007)	Nasopharynx	T1-T2b, N0-N1	IMRT: 28	Observer-rated xerostomia at 1-year	RTOG/EORTC	6-item XQ
			2D-RT: 28			
Nutting (2011)	Oro-hypopharynx	T1-T4, N0-N3	IMRT: 47	Observer-rated xerostomia at 1-year	LENT/SOMA & RTOG/EORTC	QLQ C30 & HN35 and Modified XQ
			2D-RT: 47			
Gupta (2012) #Rathod (2013)	Oro-hypopharynx, Larynx	T1-T3, N0-N2b	IMRT: 32	Physician-rated xerostomia (acute) within 3-months	RTOG/EORTC	QLQ C30 & HN35
			3D-RT: 28			
Peng (2012)	Nasopharynx	T1-T4, N0-N2	IMRT: 306	Overall survival at 5-years $Xerostomia	Kaplan-Meier and CTC version 3.0	Not assessed
			2D-RT: 310			
Ghosh-Laskar (2016)	Oro-hypopharynx, Larynx	T1-T3, N0-N2b	IMRT: 30	Physician-rated xerostomia (acute) at 2-months	RTOG/EORTC	QLQ C30 & HN35
			3D-RT: 29			
Bourhis (2017)	Oro-hypopharynx, Oral cavity	III/IV	IMRT: 94	Loco-regional control $Xerostomia	Kaplan-Meier and RTOG/EORTC	Not reported
			3D-RT: 94			

QOL = quality-of-life; IMRT = intensity-modulated radiation therapy; RT = radiotherapy; 2D = two-dimensional; 3D = three-dimensional; QLQ = quality-of-life questionnaire; HN = head-neck; RTOG = Radiation Therapy Oncology Group; EORTC = European Organization for Research and Treatment of Cancer; XQ = xerostomia questionnaire; LENT/SOMA = late effects of normal tissues/subjective objective management analytic; CTC = common toxicity criteria

*Updated outcome data from the index trial later reported and published in abstract form

^#^QOL outcome data from the index trial reported and published separately

^$^Xerostomia was assessed as a secondary endpoint

**Table 2:** Summary of clinical outcomes of studies included in the systematic review and meta-analyses.

Author (year)	Median follow-up	Technique of RT	Proportion with moderate to severe xerostomia					Loco-regional control (LRC)	Overall survival (OS)
			≤3-month¤	6-month	1-year	2-year	3-year		
Pow (2006) *Kwong (2008)	54 months	IMRT	80%	**65%**	**50%**	NK/NA	NK/NA	90.5% (4-year)	No significant difference in OS
		2D-RT	85%	**95%**	**95%**			71.7% (4-year)	
Kam (2007)	NK/NA	IMRT	**46.4%**	75%	**39.3%**	NK/NA	NK/NA	Only one local failure in each arm	NK/NA
		2D-RT	**85.7%**	92.9%	**82.1%**				
Nutting (2011)	44 months	IMRT	**63%**	**58%**	**38%**	**29%**	NK/NA	78% (2-year)	78% (2-year)
		2D-RT	**82%**	**84%**	**74%**	**84%**		80% (2-year)	76% (2-year)
Gupta (2012) #Rathod (2013)	40 months	IMRT	**59%**	**31%**	**28%**	**21%**	**0%**	80.5% (3-year)	68.0% (3-year)
		3D-RT	**89%**	**77%**	**73%**	**59%**	**56%**	88.2% (3-year)	70.6% (3-year)
Peng (2012)	42 months	IMRT	**28.1%**	**9.5%**				**90.5% (5-year)**	**79.6% (5-year)**
		2D-RT	**57.5%**	**29.7%**				**84.7% (5-year)**	**67.1% (5-year)**
Ghosh-Laskar (2016)	70 months	IMRT	**24%**	**8%**	**10%**	**0%**	**0%**	69.2% (5-year)	63.4% (5-year)
		3D-RT	**54%**	**46%**	**24%**	**22%**	**16%**	62.9% (5-year)	50.7% (5-year)
Bourhis (2017)	55 months	IMRT	Better in IMRT arm	NK/NA	**19%**	NK/NA	**8%**	3-year hazard ratio for LRC = 0.88	No significant difference in OS
		3D-RT			**66%**		**47%**		

RT = radiotherapy; IMRT = intensity-modulated radiation therapy; 2D = two-dimensional; 3D = three-dimensional; NK/NA = not known/not available

*Updated outcome data from the index trial reported and published later in abstract form

^#^Quality-of-life outcome data from the index trial reported and published separately

All significant results (p≤0.05) are highlighted in bold

### Data synthesis and meta-analysis

**Fig 2:**
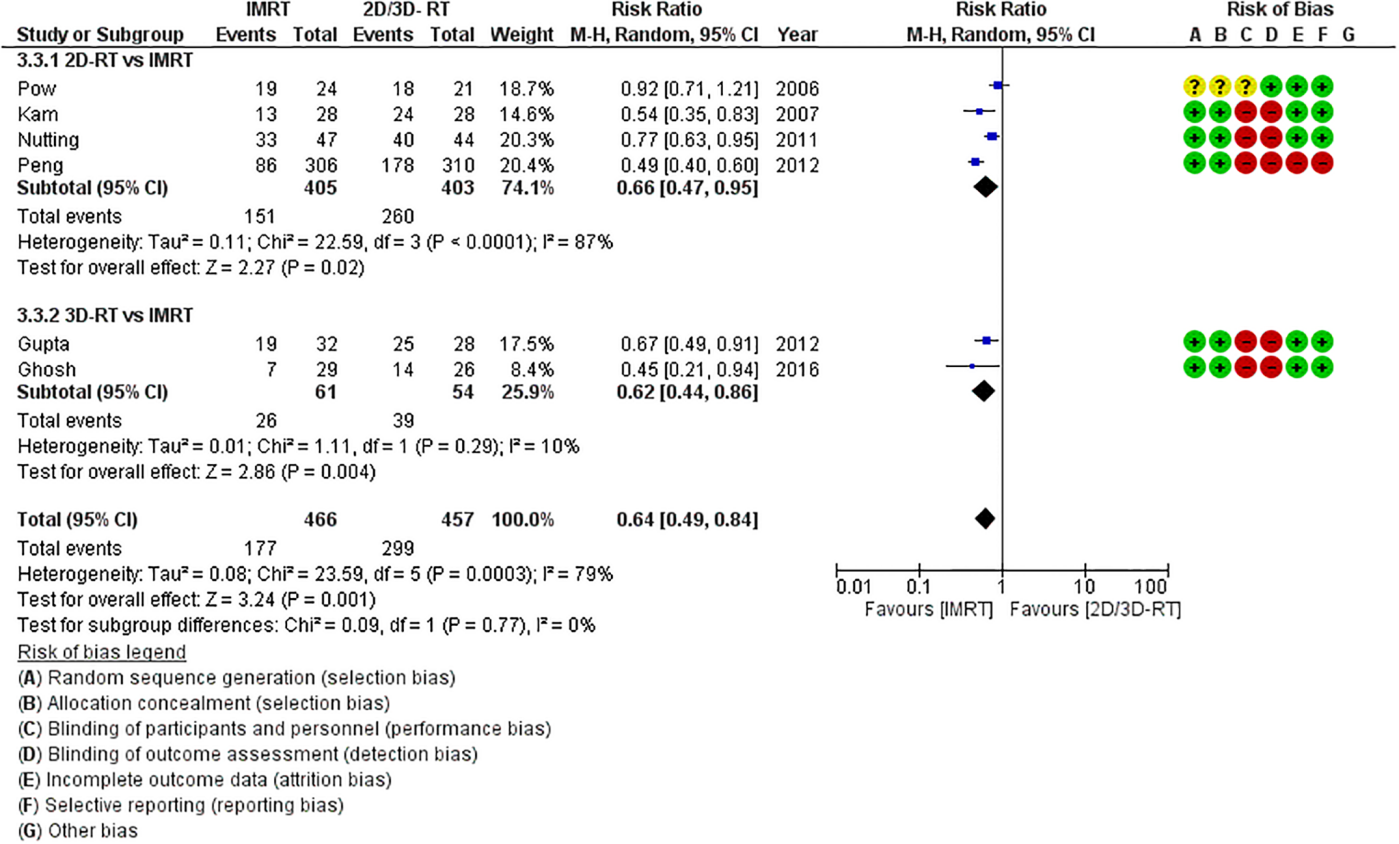
Forest plot (including the risk of bias assessment) demonstrating significant reduction in the risk of acute grade 2 or worse xerostomia with intensity modulated radiation therapy (IMRT) compared to conventional techniques. Note comparable benefit of IMRT over two-dimensional radiotherapy (2D-RT) and three-dimensional radiotherapy (3D-RT) on subgroup analyses.

**Fig 3:**
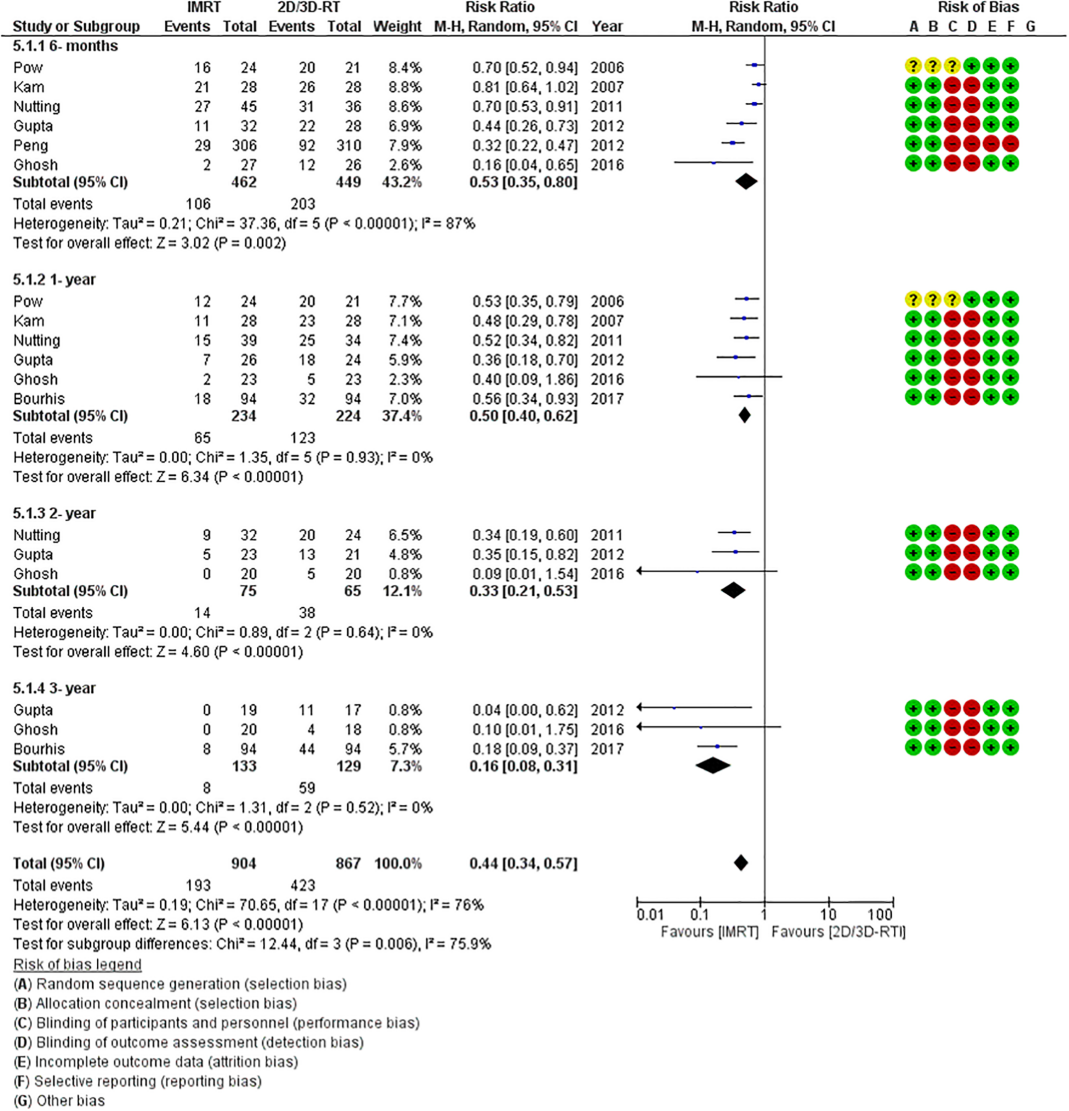
Forest plot (including the risk of bias assessment) demonstrating significant reduction in the risk of late grade 2 or worse xerostomia with intensity modulated radiation therapy (IMRT) compared to two-dimensional/three-dimensional radiotherapy (2D/3D-RT). Note the consistent and persistent benefit of IMRT over time on subgroup analyses.

There was modest heterogeneity in the included trials allowing statistical pooling of results. The primary outcome of interest i.e. xerostomia was reported in all included studies. All 7 studies reported significant reduction in moderate to severe acute xerostomia (during or within 3-months of completion of RT). The use of IMRT was associated with a 36% relative risk reduction in grade 2 or worse acute xerostomia (RR = 0.64, 95%CI = 0.49–0.84; p = 0.001) compared to 2D/3D-RT (Fig 2). On subgroup analyses stratified by technique of irradiation (Fig 2), the magnitude of benefit with IMRT over 2D-RT (RR = 0.66, 95%CI = 0.47–0.95; p = 0.02) was comparable to its benefit over 3D-RT (RR = 0.62, 95%CI = 0.44–0.86; p = 0.004). More importantly, IMRT significantly reduced the risk of late grade 2 or worse xerostomia (RR = 0.44, 95%CI = 0.34–0.57; p = 0.00001) compared to non-IMRT techniques (Fig 3). Given the expected gradual recovery of xerostomia over time, data on late xerostomia was also extracted and pooled at different time-points as previously described. The risk of grade 2 or worse xerostomia was consistently and significantly reduced with IMRT at all defined time-points (Fig 3). This reduction is late grade 2 or worse xerostomia with IMRT remained statistically significant even on subgroup analyses comparing IMRT with either 2D-RT or 3D-RT.

**Fig 4:**
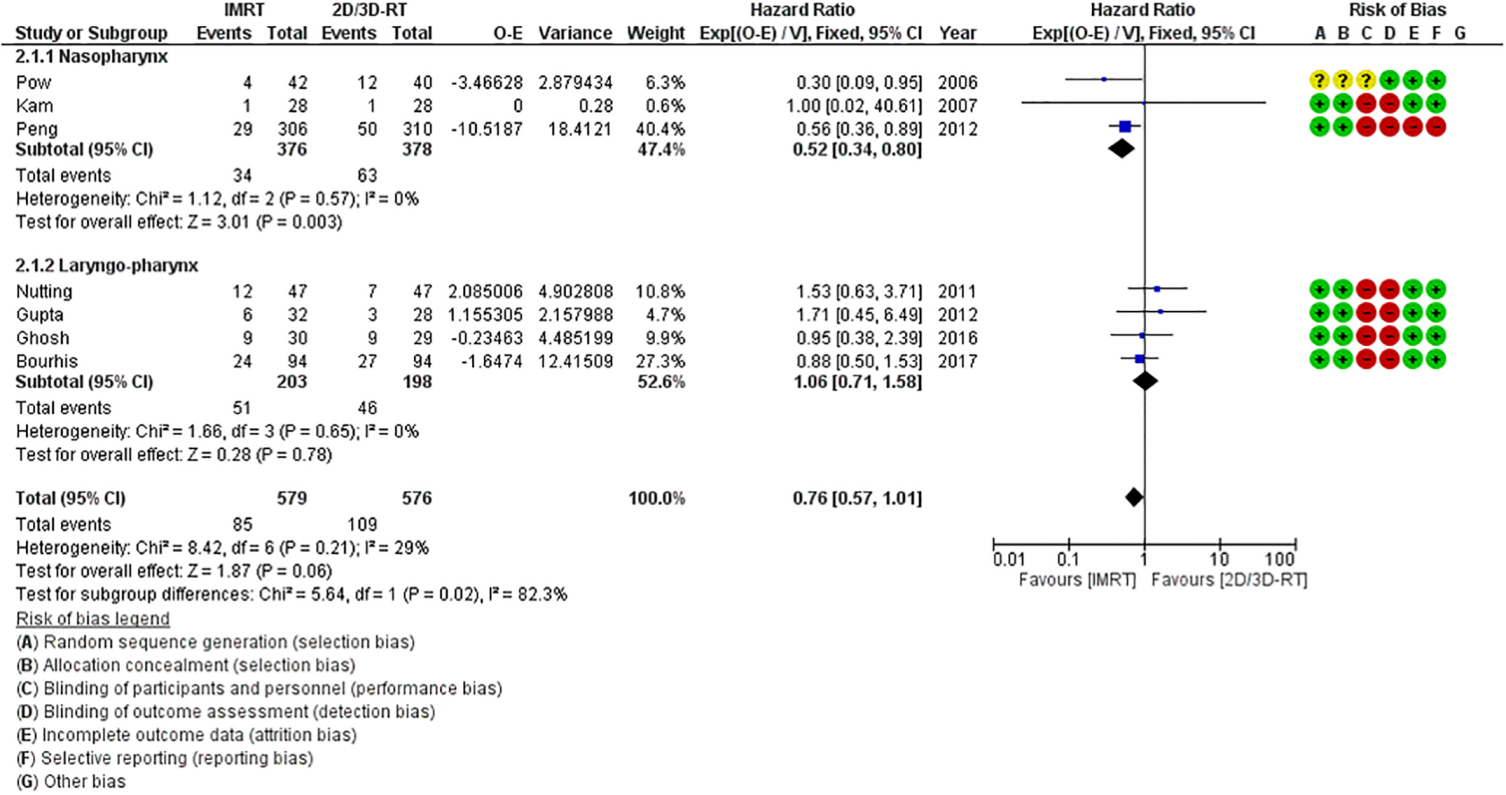
Forest plot (including the risk of bias assessment) demonstrating non-significant reduction in the risk of loco-regional relapse with intensity modulated radiation therapy (IMRT) compared to two-dimensional/three-dimensional radiotherapy (2D/3D-RT). On subgroup analyses, patients with nasopharyngeal cancers demonstrate significantly improved loco-regional control (LRC) with IMRT compared to conventional techniques, while there is no significant difference in LRC between IMRT and 2D/3D-RT for patients with cancers of the laryngo-pharynx.

All 7 RCTs presented Kaplan-Meier curves and/or reported HRs for local and regional disease control allowing data extraction and pooling for loco-regional failures. Overall, the use of IMRT was associated with a 24% relative reduction (HR = 0.76, 95%CI = 0.57–1.01) in the risk of loco-regional relapse compared to 2D/3D-RT (Fig 4), which was statistically of borderline significance (p = 0.06) as the upper bound of the 95%CI was just touching the line of unity. However, there were notable differences in LRC between IMRT and 2D/3D-RT on subgroup analyses stratified by site of primary tumor, suggesting that impact may be dependent upon primary site. For nasopharyngeal cancers, use of IMRT resulted in a very significant (48%) relative reduction in the risk of loco-regional failure (HR = 0.52, 95%CI = 0.34–0.80; p = 0.003) compared to non-IMRT techniques (Fig 4). Conversely, for cancers arising in the laryngo-pharynx, there was no significant difference in LRC between IMRT and 2D/3D-RT (HR = 1.06, 95%CI = 0.71–1.58; p = 0.78) (Fig 4).

**Fig 5:**
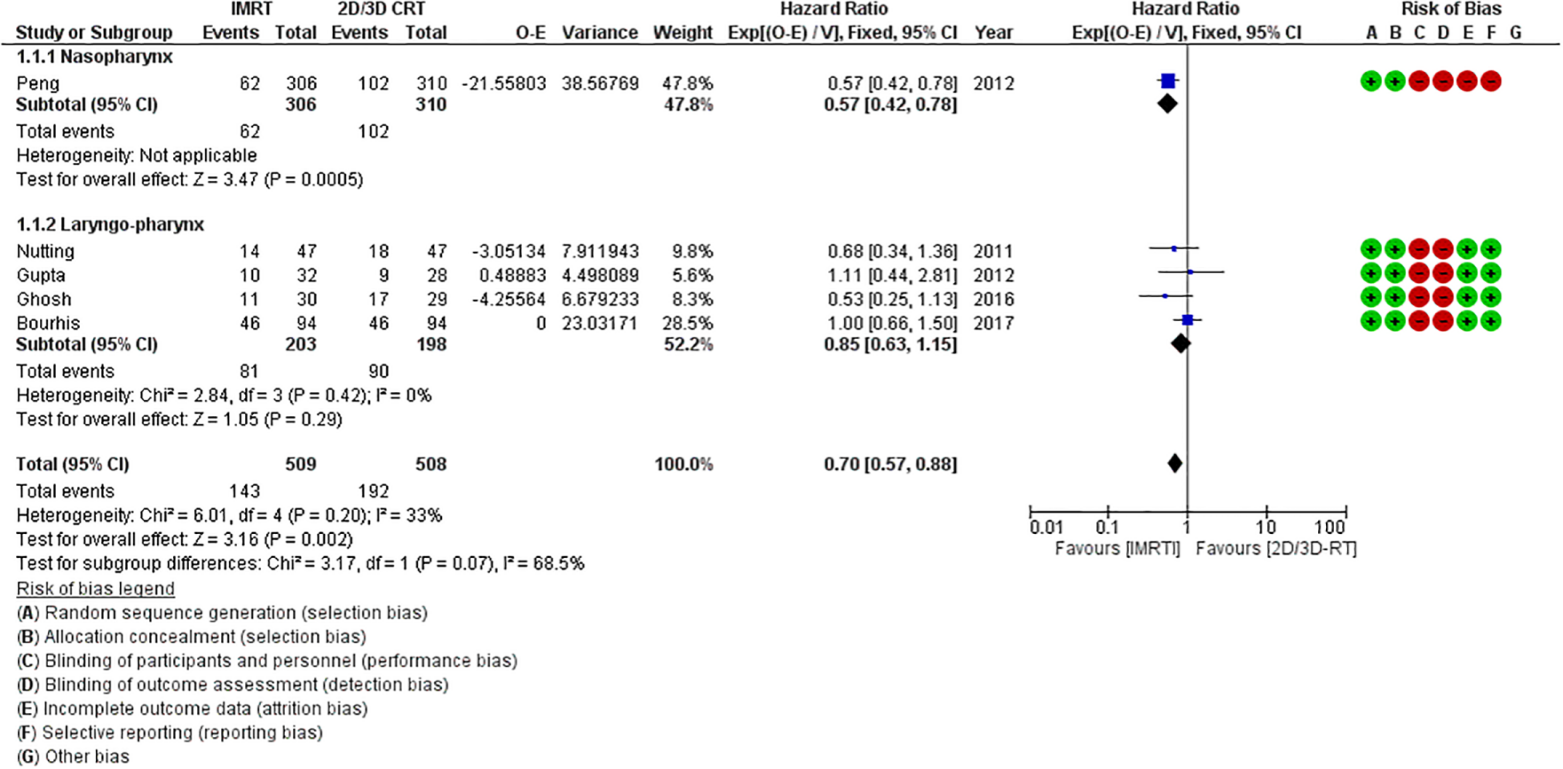
Forest plot (including the risk of bias assessment) demonstrating significant reduction in the risk of death with intensity modulated radiation therapy (IMRT) compared to two-dimensional/three-dimensional radiotherapy (2D/3D-RT). On subgroup analyses, patients with nasopharyngeal cancers demonstrate significantly improved overall survival (OS) with IMRT compared to conventional techniques, while there is no significant difference in OS between IMRT and 2D/3D-RT for patients with cancers of the laryngo-pharynx.

Five of the 7 RCTs provided extractable data on survival. In general, the use of IMRT was associated with a 30% relative reduction in the risk of death (HR = 0.70, 95%CI = 0.57–0.88; p = 0.002). However, this benefit of IMRT was again mostly dependent on primary tumor site and clearly driven by the large Chinese trial in carcinoma nasopharynx (HR = 0.57, 95%CI = 0.42–0.78; p = 0.0005). For patients with laryngo-pharyngeal caners, the use of IMRT was associated with a non-significant reduction in the risk of death (HR = 0.85, 95%CI = 0.63–1.15; p = 0.29) compared to 2D/3D-RT (Fig 5).

Although included as an endpoint in 5 RCTs, comparison of health-related QOL outcomes between IMRT and 2D/3D-RT was reported in 4 studies including one study that analyzed longitudinal evolution of QOL over time in both arms combined. There was substantial deterioration in most QOL domains immediately following RT which gradually improved over time [26]. There was consistent, gradual, though partial recovery of salivary function over time, which was significantly higher with IMRT than 2D-RT/3D-RT [15–17,26]. Xerostomia-related QOL scores were also significantly better preserved with IMRT [15–18] although global QOL was not very different [15,17] between IMRT and conventional techniques (2D/3D-RT). Lack of extractable data from most studies precluded any statistical pooling of QOL outcomes. Sensitivity analysis for all relevant outcomes of interest demonstrated lack of influence of any single study on the overall magnitude and direction of effect, interpretation, and conclusions (S1 Fig). A relatively symmetric funnel plot ruled out the presence of any significant publication bias in the weighted pooled meta-analysis (S2 Fig).

#### Strength of recommendation

The quality of evidence and strength of recommendation for all the outcome measures is summarized in S1 Table. Pursuant to the inclusion of RCTs with low or unclear risk of bias for the endpoint of xerostomia, the quality of evidence-base regarding the superiority of IMRT over conventional techniques in reducing moderate to severe acute and late xerostomia was graded as moderately high, implying that further research was unlikely to change confidence in the magnitude and direction of effect. However, evidence regarding the superiority of IMRT over 2D/3D-RT for LRC and OS was judged to be of low-quality, given the moderate to high-risk of bias for efficacy-related endpoints in the included studies coupled with inconsistency of results between the subgroups stratified on primary site (nasopharynx and laryngo-pharynx).

## Discussion

Over the last several decades, RT combined with concurrent systemic chemotherapy has become an integral component [1,2] in the curative-intent management of HNSCC, both in the definitive, non-surgical as well as post-operative adjuvant setting. Conventional techniques although capable of delivering tumoricidal doses, resulted in unintentional and unwarranted high-dose irradiation of surrounding normal critical structures situated in the vicinity of target tissues, resulting in undesirable acute as well as late toxicity [3,4] with potential negative impact upon health-related QOL [5,6]. Modern advances in treatment planning and delivery, particularly IMRT, has revolutionized contemporary oncologic practice with its potential to tightly conform high-doses to target tissues [7] with resultant better sparing of OARs such as salivary glands, uninvolved mucosa, spinal cord, brainstem, and optic pathway.

The current practice of head-neck IMRT has significantly evolved over the years. Initial dosimetric comparisons, mono-institutional single arm studies, as well as prospective multi-centric co-operative group trials [8–13] demonstrated at least comparable efficacy outcomes (LRC and OS) and consistently favorable late toxicity profile (particularly xerostomia) with IMRT compared to conventional techniques for almost all sites in the head and neck leading to its widespread adoption in routine clinical practice. The most robust evidence for the use of IMRT comes from the 7 RCTs [15–21] directly comparing IMRT with either 2D-RT or 3D-RT for various sites in the head and neck. However, most of them included relatively small number of patients and were not adequately powered for efficacy outcomes, necessitating quantitative weighted pooling of results. All the included RCTs demonstrated significant reduction in the incidence of moderate to severe acute xerostomia and consistent, gradual recovery of salivary function over time with the use of parotid-sparing IMRT with resultant favourable impact upon xerostomia-related symptoms and QOL. However, the impact upon overall and/or global QOL has been somewhat inconsistent, with most studies reporting no significant differences between IMRT and conventional techniques. Prior literature reviews [32,33] that included non-randomized observational studies in addition to the RCTs published till then have also provided contradictory conclusions regarding the impact of IMRT on overall health-related QOL.

This weighted-pooled analysis using modern meta-analytic methods provides moderate-quality evidence establishing the superiority of IMRT over 2D-RT/3D-RT for grade 2 or worse xerostomia at all time-points in the curative-intent radiotherapeutic management of HNSCC. This significant reduction in moderate to severe xerostomia with IMRT also translated into an improvement in xerostomia-specific QOL. Unfortunately, QOL data could not be pooled in this meta-analysis to provide any quantitative estimate of the impact of IMRT on QOL outcomes. The impact of IMRT for disease-related outcomes (LRC and OS) was heavily dependent upon site of primary tumor in this analysis. Patients with nasopharyngeal cancers benefitted maximally with IMRT; while there was no significant difference between IMRT and 2D/3D-RT for LRC and OS in patients with cancers of the laryngo-pharynx. Future studies specifically designed and powered to test the benefit of IMRT over 2D/3D-RT for LRC and OS would provide more conclusive evidence for disease-related outcomes. Nonetheless, this non-inferiority of IMRT for disease-related outcomes is reassuring in that the highly significant reduction in xerostomia is not occurring at the cost of disease control. However, it should be borne in mind that aggressive and overzealous sparing of parotid glands during IMRT can result in increased risk of marginal failures thereby negating any potential improvement in LRC. It assumes greater significance in the context of human papilloma virus (HPV) associated oropharyngeal cancer [34] which has now emerged worldwide as a biologically distinct subset of HNSCC with favourable prognosis. Given the expected long-term survival in HPV-associated oropharyngeal cancer, making them more vulnerable to late effects of treatment, the head-neck oncology community has been testing various strategies for de-escalation/de-intensification [35] of treatment in that subset.

### Strengths and limitations

The present meta-analysis is based only on RCTs directly comparing IMRT with either 2D-RT or 3D-RT in HNSCC identified from the medical literature using a validated search strategy. Although there was some heterogeneity across included studies, they were quite similar in terms of study design, methodology, analyses, and reporting. Appropriate subgroup analysis was done after stratifying on technique of irradiation (2D-RT or 3D-RT) and site of primary tumor (nasopharynx or laryngo-pharynx). Data on xerostomia was also pooled at later time-points to ascertain whether the significant benefit of reduction in the risk of moderate to severe acute xerostomia with IMRT persisted over time. The quality of included studies was judged to be moderately high with low to unclear risk of bias for xerostomia (primary outcome measure). However, the quality of studies for disease-related outcomes was downgraded due to high-risk of bias. Notwithstanding, disease-related outcomes were also compared providing useful information on the efficacy of IMRT for HNSCC in the curative-setting. No significant publication bias was detected for any of the outcome measures in this analysis. However, certain caveats and limitations remain. Due to lack of complete reporting in index RCTs, this meta-analysis did not attempt any comparison between IMRT and 2D/3D-RT for other significant acute toxicities of comprehensive head-neck irradiation (apart from xerostomia) such as mucositis, dermatitis, and dysphagia/odynophagia. Although xerostomia remains the most debilitating long-term toxicity of radio(chemo)therapy, chronic dysphagia can also negatively impact health-related QOL in survivors [4,6]. This meta-analysis could not assess the impact of IMRT on late dysphagia as it was not an endpoint in any of the included primary studies. An ongoing RCT that compares dysphagia aspiration related structures (DARS)-sparing and dysphagia-optimized IMRT with standard IMRT should provide definitive answers [36]. Furthermore, this meta-analysis, could not quantify QOL difference, if any, between the two techniques due to lack of easily and readily extractable or available data. It is widely accepted that IMRT is associated with significantly higher cost and complexity, compared to conventional techniques. However, none of the trials included any analyses of cost-effectiveness of IMRT, precluding any such estimation in the meta-analysis. The time-frame of included studies in the meta-analyses was variable with potential differences in quality of RT; advances in dose calculation and heterogeneity correction algorithms over time could have further improved disease control and reduced toxicity, irrespective of RT delivery technique. Finally, individual patient data was not available for pooling in this meta-analysis which was based primarily on summary data extracted from the published medical literature.

## Conclusions and Relevance

There is consistent moderate-quality evidence that IMRT significantly reduces the risk of moderate to severe acute and late xerostomia compared to 2D/3D-RT in curative-intent radiotherapeutic management of HNSCC. However, the quality of evidence regarding the superiority of IMRT over conventional techniques for disease-related outcomes (LRC and OS) is rather low due to relative lack of power and inconsistency in results of subgroup analyses stratified by primary site precluding robust conclusions.

## Supporting information

S1 Appendix Literature search strategy used in the systematic review and meta-analyses. (DOCX)

S1 Fig Sensitivity analysis demonstrating no significant influence of any individual study in the meta-analyses on the overall effect for all the outcome measures viz. acute xerostomia (a), late xerostomia (b), loco-regional control (c), and overall survival (d). (DOCX)

S2 Fig Relatively symmetric funnel-plot suggesting lack of any significant publication bias in the meta-analyses for all the outcome measures viz. acute xerostomia (a), late xerostomia (b), loco-regional control (c), and overall survival (d). (DOCX)

S1 PRISMA Checklist Statement of the presence and location of individual items of PRISMA checklist in the systematic review and meta-analyses. (DOC)

S1 Table Summary of findings table with quality of evidence and strength of recommendation for two-dimensional/three-dimensional radiotherapy (2D/3D-RT) vs intensity modulated radiation therapy (IMRT) in head and neck squamous cell carcinoma (HNSCC). (DOCX)
