# Supplementary material for: Systematic review and meta-analyses of intensity-modulated radiation therapy versus conventional two-dimensional and/or or three-dimensional radiotherapy in curative-intent management of head and neck squamous cell carcinoma
Source: PLoS One. 2018 Jul 6;13(7):e0200137. doi: 10.1371/journal.pone.0200137 (PMC6034843; doi:10.1371/journal.pone.0200137)
Supplement: S1 Appendix — (DOCX) [file pone.0200137.s001.docx]

**S1 Appendix**

**Literature search strategy**

| 1. Search head and neck neoplasms |
| --- |
| 1. Search head and neck cancer |
| 1. Search oral cancer |
| 1. Search head and neck squamous cell carcinoma |
| 1. Search HNSCC |
| 1. Search HNC |
| 1. Search SCCHN |
| 1. Search oral neoplasms |
| 1. Search mouth neoplasms |
| 1. Search cancers of mouth |
| 1. Search [(#1 OR #2 OR #3 OR #4 OR #5 OR #6 OR #7 OR #8 OR #9 OR #10 )] |
| 1. Search conventional radiotherapy |
| 1. Search conformal radiotherapy |
| 1. Search two-dimensional radiotherapy |
| 1. Search three-dimensional radiotherapy 2. Search intensity modulated radiation therapy |
| 1. Search [(2D-RT OR 3D-RT OR CRT OR 3D-CRT OR IMRT)] |
| 1. Search (#12 OR #13 OR #14 OR #15 OR #16 OR#17) |
| 1. Search conventional rad* |
| 1. Search conformal rad* |
| 1. Search precision rad* |
| 1. Search high-precision rad* |
| 1. Search (#19 OR #20 OR #21 OR #22) |
| 1. Search ([#11 AND (#18 OR #23)] AND Humans[Mesh]) |
| 1. Search ([#11 AND (#18 OR #23)] AND Humans[Mesh]) Filter: Randomized Controlled Trial |
