# Supplementary material for: Systematic review and meta-analyses of intensity-modulated radiation therapy versus conventional two-dimensional and/or or three-dimensional radiotherapy in curative-intent management of head and neck squamous cell carcinoma
Source: PLoS One. 2018 Jul 6;13(7):e0200137. doi: 10.1371/journal.pone.0200137 (PMC6034843; doi:10.1371/journal.pone.0200137)
Supplement: S1 Fig — (DOCX) [file pone.0200137.s002.docx]

**S1 Fig: Sensitivity analysis demonstrating no significant influence of any individual study in the meta-analyses on the overall effect for all the outcome measures viz. acute xerostomia (a), late xerostomia (b), loco-regional control (c), and overall survival (d)**

0.45

0.64

0.49

0.84

0.90

**Pow**

**Kam**

**Nutting**

**Peng**

**Gupta**

**Ghosh**

1. **Sensitivity Analysis for Acute Grade 2 or worse Xerostomia**

**Lower CI Limit**

**Estimate**

**Upper CI Limit**

**Risk Ratio**

0.38

0.50

0.40

0.62

0.65

**Pow**

**Kam**

**Nutting**

**Bourhis**

**Gupta**

**Ghosh**

**(b) Sensitivity Analysis for Late (1-year) Grade 2 or worse Xerostomia**

**Lower CI Limit**

**Estimate**

**Upper CI Limit**

**Risk Ratio**

**0.51**

**0.76**

**0.57**

**1.01**

**1.36**

**Peng**

**Nutting**

**Gupta**

**Ghosh**

**Bourhis**

**Pow**

**Kam**

**(c) Sensitivity Analysis for Loco-Regional Control (LRC)**

**Lower CI Limit**

**Estimate**

**Upper CI Limit**

**Study omitted**

**Hazard Ratio**

**Study omitted**

**0.47**

**0.70**

**0.56**

**0.87**

**1.15**

**Peng**

**Nutting**

**Gupta**

**Ghosh**

**Bourhis**

**(d) Sensitivity analysis for Overall Survival**

**Lower CI Limit**

**Estimate**

**Upper CI Limit**

**Hazard Ratio**
