# Supplementary material for: Systematic review and meta-analyses of intensity-modulated radiation therapy versus conventional two-dimensional and/or or three-dimensional radiotherapy in curative-intent management of head and neck squamous cell carcinoma
Source: PLoS One. 2018 Jul 6;13(7):e0200137. doi: 10.1371/journal.pone.0200137 (PMC6034843; doi:10.1371/journal.pone.0200137)

**S2 Fig: Relatively symmetric funnel plot suggesting lack of any significant publication bias in the meta-analyses for all the outcome measures viz. acute xerostomia (a), late xerostomia (b), loco-regional control (c), and overall survival (d)**

1. **Acute Xerostomia**


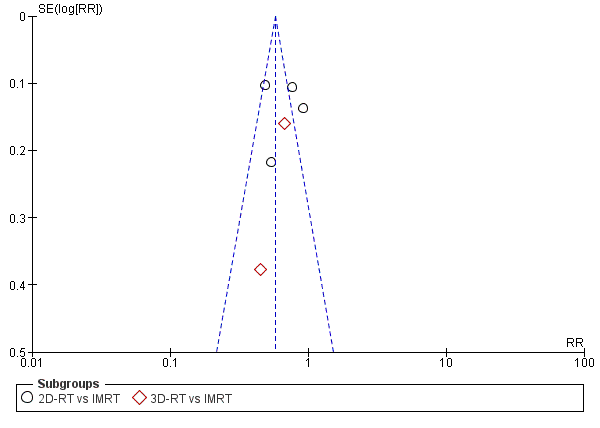


1. **Late Xerostomia**


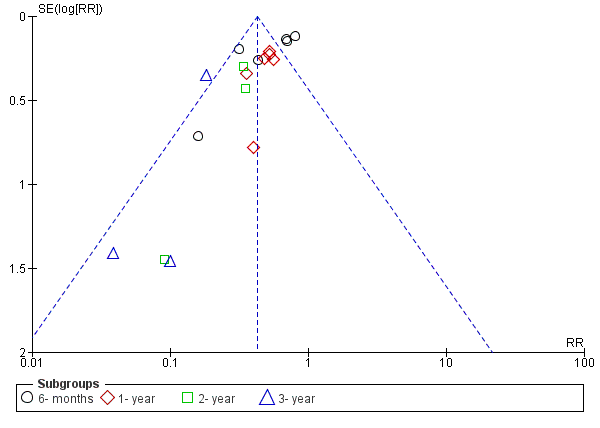


1. **Loco-Regional Control**


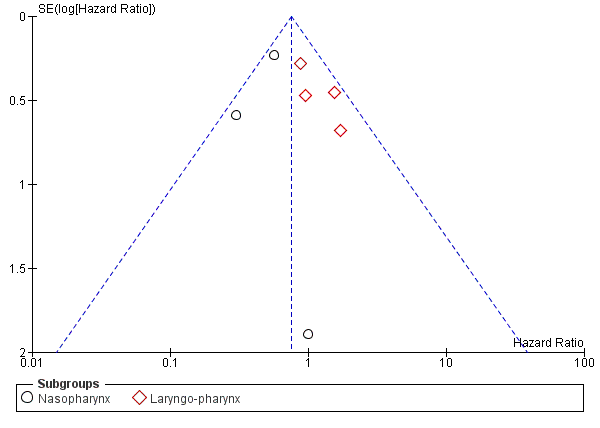


1. **Overall Survival**


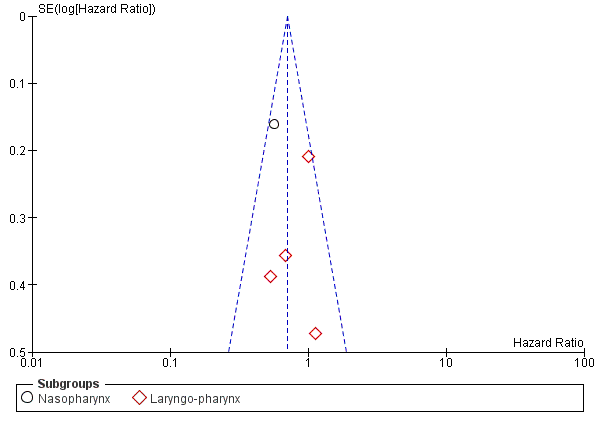

Supplement: S2 Fig — (DOCX) [file pone.0200137.s003.docx]
