# Supplementary material for: Systematic review and meta-analyses of intensity-modulated radiation therapy versus conventional two-dimensional and/or or three-dimensional radiotherapy in curative-intent management of head and neck squamous cell carcinoma
Source: PLoS One. 2018 Jul 6;13(7):e0200137. doi: 10.1371/journal.pone.0200137 (PMC6034843; doi:10.1371/journal.pone.0200137)
Supplement: S1 Table — (DOCX) [file pone.0200137.s005.docx]

**S1 Table: Summary of findings table with quality of evidence and strength of recommendation for two-dimensional/three-dimensional radiotherapy (2D/3D-RT) vs intensity-modulated radiation therapy (IMRT) in head and neck squamous cell carcinoma (HNSCC)**

| **2D/3D-RT versus IMRT for HNSCC: Acute grade 2 or worse Xerostomia** | | | | | |
| --- | --- | --- | --- | --- | --- |
| **Outcomes** | **No of Participants (studies)** Follow up | **Quality of the evidence** (GRADE) | **Relative effect (95% CI)** | **Anticipated absolute effects** | |
|  |  |  |  |  | |
|  |  |  |  | **Risk with Control** | **Risk difference with Acute >= grade 2 Xerostomia** (95% CI) |
| **All Studies** | 923 (6) |  | **RR 0.57**  (0.5 to 0.65) | **Study population** | |
|  |  |  |  | **654 per 1000** | **281 fewer per 1000** (from 229 fewer to 327 fewer) |
|  |  |  |  | **Moderate** | |
|  |  |  |  | **857 per 1000** | **369 fewer per 1000** (from 300 fewer to 428 fewer) |
| **2D-RT vs IMRT** | 808 (4 studies) | ⊕⊕⊕⊝ **MODERATE**^1^ due to risk of bias | **RR 0.57**  (0.5 to 0.66) | **Study population** | |
|  |  |  |  | **645 per 1000** | **277 fewer per 1000** (from 219 fewer to 323 fewer) |
|  |  |  |  | **Moderate** | |
|  |  |  |  | **857 per 1000** | **369 fewer per 1000** (from 291 fewer to 428 fewer) |
| **3D-RT vs IMRT** | 115 (2 studies) | ⊕⊕⊕⊝ **MODERATE**^2^ due to risk of bias | **RR 0.59**  (0.43 to 0.8) | **Study population** | |
|  |  |  |  | **722 per 1000** | **296 fewer per 1000** (from 144 fewer to 412 fewer) |
|  |  |  |  | **Moderate** | |
|  |  |  |  | **716 per 1000** | **294 fewer per 1000** (from 143 fewer to 408 fewer) |
| *The basis for the **assumed risk** (e.g. the median control group risk across studies) is provided in footnotes. The **corresponding risk** (and its 95% confidence interval) is based on the assumed risk in the comparison group and the **relative effect** of the intervention (and its 95% CI). **CI:** Confidence interval; **RR:** Risk ratio; | | | | | |
| GRADE Working Group grades of evidence **High quality:** Further research is very unlikely to change our confidence in the estimate of effect.  **Moderate quality:** Further research is likely to have an important impact on our confidence in the estimate of effect and may change the estimate. **Low quality:** Further research is very likely to have an important impact on our confidence in the estimate of effect and is likely to change the estimate. **Very low quality:** We are very uncertain about the estimate. | | | | | |
| ^1^ Lack of blinding. ^2^ Lack of blinding | | | | | |

| **2D/3D-RT versus IMRT for HNSCC: Late (1-year) grade 2 or worse Xerostomia** | | | | | |
| --- | --- | --- | --- | --- | --- |
| **Outcomes** | **No of Participants (studies)** Follow up | **Quality of the evidence** (GRADE) | **Relative effect (95% CI)** | **Anticipated absolute effects** | |
|  |  |  |  |  | |
|  |  |  |  | **Risk with Control** | **Risk difference with Late Xerostomia** (95% CI) |
| **Late (1-yr) ≥grade 2 Xerostomia** | 458 (6 studies) | ⊕⊕⊕⊝ **MODERATE**^1^ due to risk of bias | **RR 0.5**  (0.4 to 0.62) | **Study population** | |
|  |  |  |  | **549 per 1000** | **275 fewer per 1000** (from 209 fewer to 329 fewer) |
|  |  |  |  | **Moderate** | |
|  |  |  |  | **743 per 1000** | **372 fewer per 1000** (from 282 fewer to 446 fewer) |
| *The basis for the **assumed risk** (e.g. the median control group risk across studies) is provided in footnotes. The **corresponding risk** (and its 95% confidence interval) is based on the assumed risk in the comparison group and the **relative effect** of the intervention (and its 95% CI).  **CI:** Confidence interval; **RR:** Risk ratio; | | | | | |
| GRADE Working Group grades of evidence **High quality:** Further research is very unlikely to change our confidence in the estimate of effect.  **Moderate quality:** Further research is likely to have an important impact on our confidence in the estimate of effect and may change the estimate. **Low quality:** Further research is very likely to have an important impact on our confidence in the estimate of effect and is likely to change the estimate. **Very low quality:** We are very uncertain about the estimate. | | | | | |
| ^1^ Lack of blinding. | | | | | |

| **2D/3D-RT versus IMRT for HNSCC: Loco-regional control (LRC)** | | | | | | |
| --- | --- | --- | --- | --- | --- | --- |
| **Outcome** | **No of Participants (studies)** Follow up | **Quality of the evidence** (GRADE) | **Relative effect (95% CI)** | | **Anticipated absolute effects** | |
|  |  |  |  |  |  | |
|  |  |  |  |  | **Risk with Control** | **Risk difference with Loco-regional control** (95% CI) |
| **All Studies** | 1155 (7) |  | **HR 0.76**  (0.57 to 1.01) | | **Study population** | |
|  |  |  |  |  | **189 LRC per 1000** | **42 fewer LRC per 1000** (from 77 fewer to 2 more) |
|  |  |  |  |  | **Moderate** | |
|  |  |  |  |  | **161 LRC per 1000** | **36 fewer LRC per 1000** (from 66 fewer to 1 more) |
| **Nasopharynx** | 754 (3 studies) | ⊕⊝⊝⊝ **VERY LOW**^1,2,3^ due to risk of bias, inconsistency, imprecision | **HR 0.52**  (0.34 to 0.8) | | **Study population** | |
|  |  |  |  |  | **167 per 1000** | **76 fewer per 1000** (from 31 fewer to 107 fewer) |
|  |  |  |  |  | **Moderate** | |
|  |  |  |  |  | **161 per 1000** | **74 fewer per 1000** (from 30 fewer to 103 fewer) |
| **Laryngo-pharynx** | 401 (4 studies) | ⊕⊕⊝⊝ **LOW**^4,5^ due to risk of bias, inconsistency | **HR 1.06**  (0.71 to 1.58) | | **Study population** | |
|  |  |  |  |  | **232 per 1000** | **12 more per 1000** (from 61 fewer to 109 more) |
|  |  |  |  |  | **Moderate** | |
|  |  |  |  |  | **218 per 1000** | **11 more per 1000** (from 58 fewer to 104 more) |
| *The basis for the **assumed risk** (e.g. the median control group risk across studies) is provided in footnotes. The **corresponding risk** (and its 95% confidence interval) is based on the assumed risk in the comparison group and the **relative effect** of the intervention (and its 95% CI).  **CI:** Confidence interval; **HR:** Hazard ratio; | | | | | | |
| GRADE Working Group grades of evidence **High quality:** Further research is very unlikely to change our confidence in the estimate of effect.  **Moderate quality:** Further research is likely to have an important impact on our confidence in the estimate of effect and may change the estimate. **Low quality:** Further research is very likely to have an important impact on our confidence in the estimate of effect and is likely to change the estimate. **Very low quality:** We are very uncertain about the estimate. | | | | | | |
| ^1^ Lack of blinding and incomplete outcome data. ^2^ Similar event rates in both arms in one of three studies. ^3^ Small sample size in two studies. ^4^ Lack of blinding. ^5^ Discrepant results between the four studies. | | | | | | |
| **2D/3D-RT versus IMRT for HNSCC: Overall Survival (OS)** | | | | | | |
| **Outcomes** | **No of Participants (studies)** Follow up | **Quality of the evidence** (GRADE) | **Relative effect (95% CI)** | **Anticipated absolute effects** | | |
|  |  |  |  |  | | |
|  |  |  |  | **Risk with Control** | | **Risk difference with Overall Survival**  (95% CI) |
| **All studies** | 1017 (5) |  | **HR 0.7**  (0.57 to 0.88) | **Study population** | | |
|  |  |  |  | **378 per 1000** | | **95 fewer per 1000** (from 36 fewer to 141 fewer) |
|  |  |  |  | **Moderate** | | |
|  |  |  |  | **383 per 1000** | | **96 fewer per 1000** (from 37 fewer to 142 fewer) |
| **Nasopharynx** | 616 (1 study) | ⊕⊕⊕⊝ **MODERATE**^1,2^ due to risk of bias | **HR 0.57**  (0.42 to 0.78) | **Study population** | | |
|  |  |  |  | **329 per 1000** | | **126 fewer per 1000** (from 62 fewer to 175 fewer) |
|  |  |  |  | **Moderate** | | |
|  |  |  |  | **329 per 1000** | | **126 fewer per 1000** (from 62 fewer to 175 fewer) |
| **Laryngo-pharynx** | 401 (4 studies) | ⊕⊕⊝⊝ **LOW**^3,4^ due to risk of bias, imprecision | **HR 0.85**  (0.63 to 1.15) | **Study population** | | |
|  |  |  |  | **455 per 1000** | | **52 fewer per 1000** (from 137 fewer to 47 more) |
|  |  |  |  | **Moderate** | | |
|  |  |  |  | **436 per 1000** | | **51 fewer per 1000** (from 133 fewer to 46 more) |
| *The basis for the **assumed risk** (e.g. the median control group risk across studies) is provided in footnotes. The **corresponding risk** (and its 95% confidence interval) is based on the assumed risk in the comparison group and the **relative effect** of the intervention (and its 95% CI).  **CI:** Confidence interval; **HR:** Hazard ratio; | | | | | | |
| GRADE Working Group grades of evidence **High quality:** Further research is very unlikely to change our confidence in the estimate of effect.  **Moderate quality:** Further research is likely to have an important impact on our confidence in the estimate of effect and may change the estimate. **Low quality:** Further research is very likely to have an important impact on our confidence in the estimate of effect and is likely to change the estimate. **Very low quality:** We are very uncertain about the estimate. | | | | | | |
| ^1^ No explanation was provided; ^2^ only a single study. ^3^ Lack of blinding ^4^ Small sample size in individual studies | | | | | | |
